# Automated Foveal Avascular Zone Segmentation in Optical Coherence Tomography Angiography Across Multiple Eye Diseases Using Knowledge Distillation

**DOI:** 10.3390/bioengineering12040334

**Published:** 2025-03-23

**Authors:** Peter Racioppo, Aya Alhasany, Nhuan Vu Pham, Ziyuan Wang, Giulia Corradetti, Gary Mikaelian, Yannis M. Paulus, SriniVas R. Sadda, Zhihong Hu

**Affiliations:** 1 Doheny Image Analysis Laboratory, Doheny Eye Institute, 150 North Orange Grove Blvd, Pasadena, CA 91103, USA; 2 Doheny Image Reading and Research Laboratory, Doheny Eye Institute, 150 North Orange Grove Blvd, Pasadena, CA 91103, USA; 3 Hedgefog Research Inc., 1891 N Gaffey St. Ste 224, San Pedro, CA 90731, USA; 4 Wilmer Eye Institute, Department of Ophthalmology, Johns Hopkins University, 1800 Orleans St, Baltimore, MD 21287, USA

**Keywords:** foveal avascular zone, automated segmentation, eye diseases, imbalanced data, ocular disorders, knowledge distillation, vision transformer, OCTA, color fundus photography

## Abstract

Optical coherence tomography angiography (OCTA) is a noninvasive imaging technique used to visualize retinal blood flow and identify changes in vascular density and enlargement or distortion of the foveal avascular zone (FAZ), which are indicators of various eye diseases. Although several automated FAZ detection and segmentation algorithms have been developed for use with OCTA, their performance can vary significantly due to differences in data accessibility of OCTA in different retinal pathologies, and differences in image quality in different subjects and/or different OCTA devices. For example, data from subjects with direct macular damage, such as in age-related macular degeneration (AMD), are more readily available in eye clinics, while data on macular damage due to systemic diseases like Alzheimer’s disease are often less accessible; data from healthy subjects may have better OCTA quality than subjects with ophthalmic pathologies. Typically, segmentation algorithms make use of convolutional neural networks and, more recently, vision transformers, which make use of both long-range context and fine-grained detail. However, transformers are known to be data-hungry, and may overfit small datasets, such as those common for FAZ segmentation in OCTA, to which there is limited access in clinical practice. To improve model generalization in low-data or imbalanced settings, we propose a multi-condition transformer-based architecture that uses four teacher encoders to distill knowledge into a shared base model, enabling the transfer of learned features across multiple datasets. These include intra-modality distillation using OCTA datasets from four ocular conditions: healthy aging eyes, Alzheimer’s disease, AMD, and diabetic retinopathy; and inter-modality distillation incorporating color fundus photographs of subjects undergoing laser photocoagulation therapy. Our multi-condition model achieved a mean Dice Index of 83.8% with pretraining, outperforming single-condition models (mean of 83.1%) across all conditions. Pretraining on color fundus photocoagulation images improved the average Dice Index by a small margin on all conditions except AMD (1.1% on single-condition models, and 0.1% on multi-condition models). Our architecture demonstrates potential for broader applications in detecting and analyzing ophthalmic and systemic diseases across diverse imaging datasets and settings.

## 1. Introduction

Optical coherence tomography (OCT) is a high-resolution, noninvasive imaging technique for visualizing tissue structures. Optical coherence tomography angiography (OCTA) adds the capability to visualize blood flow and retinal vasculature. OCTA has become a preferred tool for retinal imaging due to its ability to visualize the microvasculature of the retina and choroid without the need for exogenous dye injections. OCT captures detailed 3D images of the retina, while OCTA generates en face images—2D projections of specific vascular layers within 3D images [1,2].

The retinal layers contain the superficial vascular complex (SVC) in the nerve fiber and ganglion cell layers and the deep vascular complex (DVC) in the inner nuclear and outer plexiform layers. The foveal avascular zone (FAZ) is a small, capillary-free region located at the center of the fovea, a specialized area of the retina with densely packed cone photoreceptors that enable high-resolution vision. The FAZ forms a roughly circular, shallow depression, free of blood vessels, to maximize light reaching the foveola—the center of the fovea, where cone cells are most concentrated. The FAZ presents at multiple retinal layers/complexes. The area and shape of the FAZ and the density of retinal vasculature in the surrounding area are markers of visual acuity and can also indicate the presence and progression of disease processes.

FAZ is a sensitive indicator of retinal microcirculation and ischemic events. Its enlargement signals the loss of retinal vasculature, disrupting the supply of essential nutrients and oxygen to retinal tissues, potentially causing ischemia, inflammation, and ultimately leading to retinal neovascularization. Enlargement and irregular shape of the FAZ have been observed in various diseases affecting the retina, including Alzheimer’s disease (ALZ) [3,4], diabetic retinopathy (DR) [5], age-related macular degeneration (AMD) [6], macular branch retinal vein occlusion (BRVO) [7,8], and Stargardt disease [9]. Morphological changes in the FAZ generally reduce visual acuity by lowering the efficiency of light transmission to the foveola. A decline in visual acuity has been found to correlate with enlargement of the FAZ in multiple retinal diseases [10,11]. Decreased vascular perfusion near the FAZ has been observed in age-related macular degeneration [12]. The size of the FAZ is strongly positively correlated with capillary non-perfusion in patients with diabetic retinopathy [13]. The size of the FAZ correlates with disease severity in DR and can serve as a diagnostic marker. Diabetic patients without clinically detectable DR show microvascular changes, including an enlarged FAZ, which can serve as an early indicator of DR, even before the clinical signs of the disease appear [14]. OCTA FAZ area measurements reveal early retinal microvascular changes, including neovascularization and microaneurysms, in DR patients with hypertension, preceding detection by spectral domain OCT (SD-OCT) and conventional retinography [15]. FAZ enlargement has also been noted in post-COVID patients, where underlying mechanisms such as hypoxia, impaired diffusion, and undernutrition are implicated [16]. Other conditions in which changes in the FAZ area have been observed include diabetic macular edema [17], quiescent posterior uveitis and panuveitis [18], ischemic stroke, and Behҫet’s disease [19]. Studies have identified significant decreases in the FAZ area following panretinal photocoagulation (PRP) treatment in DR patients [20], after intravitreal anti-VEGF treatment in DR patients [17,21], and after glaucoma surgery [22].

The shape of the FAZ can also be an indicator of retinal disorders. For instance, diabetic eyes, even without the presence of clinically detectable diabetic retinopathy, have been found to exhibit irregular morphology and borders, including significant differences in circularity, roundness, and solidity, and an increased axial ratio [23,24,25]. Studies have reported an increased FAZ perimeter and a decreased circularity index in glaucoma, suggesting that changes in the FAZ circularity index may reflect disruption of the parafoveal capillary network [26]. The FAZ perimeter has demonstrated strong diagnostic ability in distinguishing eyes with central visual field defects from normal eyes and may serve as a biomarker for detecting individuals with central visual field defects in glaucoma [27]. Studies have found that the circularity of the FAZ is lower in subjects with high myopia [28,29], and that it is larger and more irregular in subjects with pathological myopia [30].

The automated segmentation and analysis of the FAZ in OCTA are garnering significant interest due to their potential to improve the diagnosis, monitoring, and management of various eye diseases. In this paper, we develop segmentation models for automated detection of the FAZ in retinal images, with a focus on enhancing performance across multiple conditions (Healthy, ALZ, AMD, DR). We use multi-condition models and pretraining to improve model accuracy and generalization, particularly for small datasets, to develop more reliable diagnostic tools for retinal and systemic diseases.

## 2. Methods

### 2.1. Background

**Figure 1:**
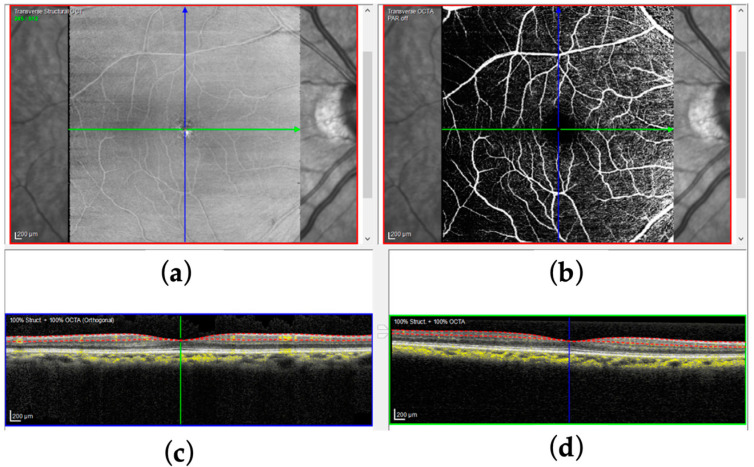
Illustration of OCT, OCTA, and FAZ. (**a**) OCT en face obtained from the SVC layer; (**b**) corresponding OCTA en face, i.e., the layer between the dotted red lines in the corresponding OCT (bottom) (Spectralis OCT2, Heidelberg Eng.). (**c**) OCT Ascan (Gray) and (**d**) OCT Bscan overlaid by OCTA signal (yellow).

**Figure 2:**
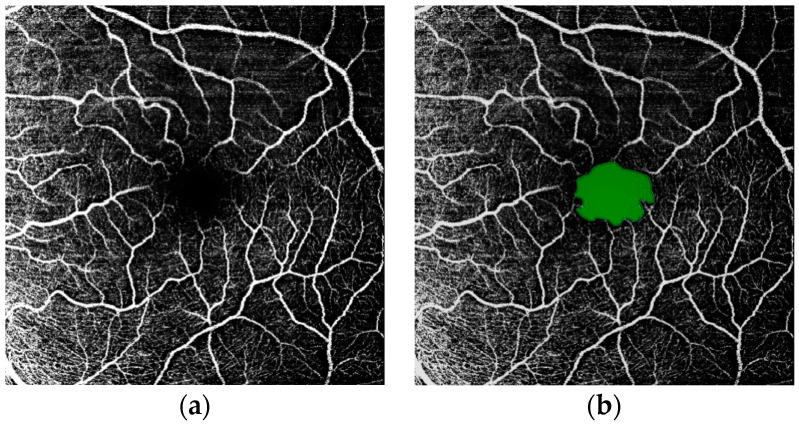
(**a**) OCTA image of the retina superficial vascular complex (SVC) in a normal eye, displaying microvessel trees surrounding the foveal avascular zone (FAZ), the capillary-free region near the center of the image. (**b**) The same image with the FAZ marked in green.

Recent advances in OCTA imaging allow for the detailed visualization of the FAZ and retinal vasculature. Figure 1 shows examples of OCT and OCTA en face images obtained from the SVC, where FAZ presents in the foveal regions in OCTA (Spectralis OCT2, Heidelberg Eng.). Figure 2 shows an annotated SVC en face map of the FAZ. Vessel density (SVC VD) can be estimated from every OCTA image (for precise definitions, see: [31]). Machine learning models have been employed for OCT image processing to improve diagnostic speed and accuracy [32], to classify retinal diseases using OCT images [33], and to accurately segment retinal layers, which is useful in diagnosing various conditions [34]. Automated segmentation of the FAZ can assist in medical diagnosis and management of various eye diseases, as discussed above [35]. Several machine learning methods have been proposed for characterizing the FAZ and surrounding vasculature in the OCTA en face maps of the macular region. These include a method based on gradient boosting to diagnose ALZ using features extracted from FAZ segmentations and convolutional neural networks (CNNs) for automated segmentation [36,37]. U-Net, a CNN-based model commonly used for image segmentation, particularly in biomedical applications, has been employed for segmentation of the FAZ and surrounding vasculature [38,39]. A 2022 study proposed a modified U-Net for FAZ segmentation, which utilizes attention modules in the skip connections so the model can better integrate features at different scales [40]. A segmentation pipeline based on Mask R-CNN was also proposed [41,42].

More recently, vision transformers (ViTs) have achieved state of the art performance on various vision tasks through the use of self-attention, rather than convolution, which allows them to efficiently aggregate information globally, capture rich contextual relationships, and dynamically adjust the region of focus depending on the input [43,44]. ViTs have achieved competitive performance on semantic segmentation tasks including in automated segmentation of retinal lesions in OCT images [45,46]. ViTs’ greater generality enables them to often outperform CNNs on large datasets [47]. Conversely, the strong inductive bias conferred by the convolution operation in CNNs biases the latter toward learning local spatial relationships and hierarchical structure in images, which helps them learn relevant features with fewer parameters and generalize well from smaller datasets [47]. The Swin Transformer addresses some of the problems with the original ViT by incorporating a shifted window attention mechanism and a hierarchical design that progressively reduces spatial dimensions while increasing feature depth (much like in U-Net) [48].

Biomedical imaging data are often scarce due to the high costs of data acquisition and annotation, the need for specialized expertise, and patient privacy concerns [49]. Deep neural networks trained on small datasets are prone to overfitting, leading to poor generalization. In many medical imaging datasets, some data classes have significantly more instances. Data imbalance can pose significant challenges, including bias towards the majority classes, negative knowledge transfer, and poor generalization [50,51,52,53]. Disparities in data accessibility hence affect both the training of deep learning models and their clinical applicability. This is compounded by challenges such as visual artifacts and variability inherent in retinal imaging, which often complicates accurate FAZ segmentation [54].

Many techniques have been proposed to address data scarcity and imbalance [55,56]: Data augmentation improves generalization by applying transformations like rotation, flipping, or noise injection to existing samples, increasing diversity [57]. Synthetic data generation expands datasets by creating artificial samples, while semi-supervised learning leverages both labeled and unlabeled data, often through pseudo-labeling or consistency regularization [58]. Self-supervised learning generates supervisory signals from the structure or properties of unlabeled data itself, allowing models to learn meaningful representations without requiring explicit labels [59]. A common approach is to do self-supervised pretraining of large foundation models on natural images, and then fine-tune them on medical data [60]. Large datasets of radiological images, such as RadImageNet, have been created for pretraining foundation models [61]. A 2024 study found that a foundation model trained using a database of medical images from different imaging types, including tomography, microscopy, and X-ray images, improved performance over pretraining on non-medical images [62]. Balancing techniques, such as over-sampling and under-sampling, address class imbalances by adjusting the data distribution, though they risk noise or information loss [63]. Approaches such as class weighting prioritize minority-class performance but require careful tuning to prevent overcompensation [64].

Ensemble methods improve robustness by combining predictions from multiple models [65]. Transfer learning enables effective training on limited data by fine-tuning pre-trained models from large, related datasets [60]. Transfer learning techniques allow us to integrate shared information across different diseases and can improve performance on under-represented data. While transfer learning adapts knowledge across tasks, model distillation or knowledge distillation addresses challenges posed by scarce and unbalanced data by allowing a larger, well-trained teacher model to impart knowledge to a smaller student model, enabling it to generalize better and make more accurate predictions despite limited or imbalanced training samples [52,53]. While transfer learning retains the original model’s architecture, leveraging its learned features, knowledge distillation results in a compressed model that retains much of the performance of the larger model while being faster and more efficient. Studies have shown that model distillation can also help mitigate overfitting [53]. Model distillation has been shown to improve model efficiency on small medical image datasets [66,67]. Qin et al. introduce one such architecture, which enhances a medical image segmentation model via knowledge distillation, enabling the smaller network to better represent the differences between tissue regions [68]. Xing et al. apply knowledge distillation to improve medical image classification, and use a mean-teacher model and contrastive loss to address intra-class variance and class imbalance [69]. Du et al. propose MDViT, a multi-domain vision transformer with a universal network for shared knowledge and “peer” branches for domain-specific learning. Each self-attention block includes a “domain adapter” that adjusts attention heads based on the task, promoting head specialization [70]. Benech et al. used a deep learning model based on Mask R-CNN to show that automated segmentation of the FAZ can be improved by incorporating auxiliary tasks for superficial vascular complex FAZ (SVC FAZ) boundary and vessel segmentation [41,71].

### 2.2. Overview

**Figure 3:**
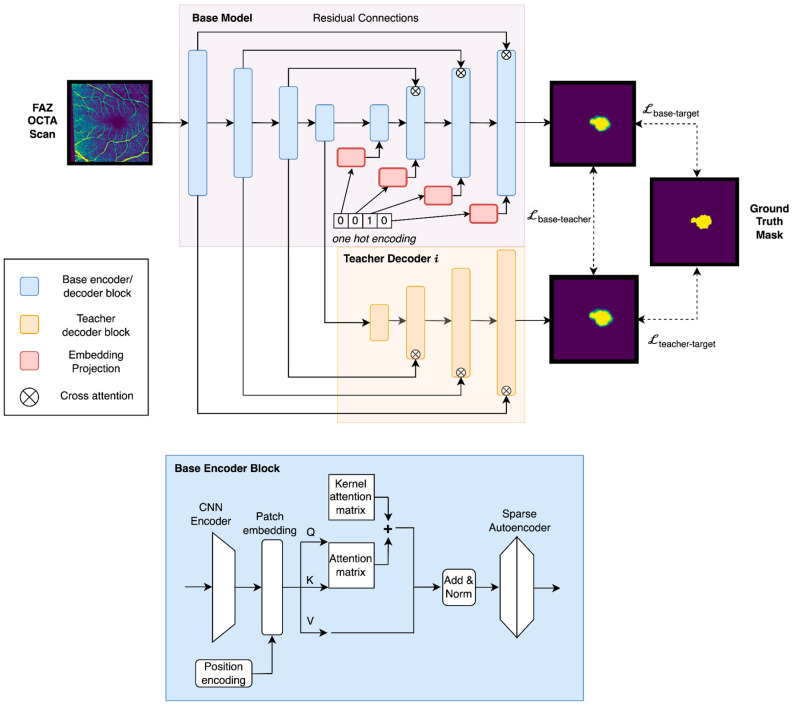
**Top:** Multi-condition model architecture with a shared Base encoder-decoder for all conditions (blue) and condition-specific Teacher decoders for each target condition (yellow). **Bottom:** Structure of a single base-encoder block, altering the standard transformer block to include a context-independent kernel attention matrix and sparse autoencoder for improved generalizability. (Decoder blocks are the same, but with a CNN Decoder placed after the multi-head attention block).

We propose a neural network architecture for FAZ segmentation that addresses both single-condition and multi-condition scenarios (Figure 3). For single-condition segmentation, the encoder-decoder backbone combines CNN and Transformer blocks to balance local feature extraction with global context modeling. Residual connections and hierarchical attention mechanisms ensure efficient feature representation and accurate reconstruction. In the multi-condition setting, condition-specific teacher decoders guide the shared backbone using knowledge distillation, enabling the base model to generalize across multiple datasets while maintaining robust performance on individual conditions. Sparse attention mechanisms are applied to improve generalization, and a hybrid loss function is used to regularize the model when working with limited data.

### 2.3. Materials

The study cohort includes 102 eyes in total from 67 patients: among them, 17 eyes (9 OS-left eye, 8 OD-right eye) from 9 elderly adults; 33 eyes (16 OS, 17 OD) from 18 patients with ALZ; 31 eyes (12 OS, 19 OD) from 28 patients with AMD; and 21 eyes (11 OS, 10 OD) from 12 patients with DR, respectively. OCTA macular images were captured with Spectralis OCT2 (Heidelberg Eng.) using scan pattern 3 mm × 3 mm (10° × 10°; 256 × 256 pixels). Both the superficial vascular complex (SVC) and deep vascular complex (DVC) may show FAZ abnormalities in the retinal diseases mentioned above; however, in this pilot study focusing on multi-domain segmentation, we concentrate on the automated segmentation of the FAZ in the SVC. The ground truth binary masks of the FAZ boundary were produced by two trained human annotators with expertise in manual grading of retinal images for clinical trial and research studies.

### 2.4. Single-Condition FAZ Segmentation

Our base model consists of alternating CNN and Transformer blocks in an encoder-decoder structure, with residual connections between the encoder and decoder. The encoder progressively reduces spatial dimensions with convolutions while increasing feature depth, and the decoder reverses this process with deconvolutions. Convolution and deconvolution layers integrate spatially local information, with lower layers capturing long-range dependencies and higher layers capturing finer details. Residual connections across layers help retain information lost in max pooling. Transformer encoder layers placed between each step of convolution and deconvolution allow image patch feature embeddings to exchange information globally. The result is a hierarchical arrangement of multi-head attention layers, with transformers in the first layers of the encoder and last layers of the decoder attending to many smaller image patches with low-dimensional embeddings, and intermediate layers attending to larger image patches with higher-dimensional embeddings. This allows attention to be applied to both more abstract representations (based on larger image patches) and more fine-grained spatial information (based on smaller image patches). Residual connections are integrated with upstream information using cross attention. A fully connected network connects the encoder and decoder.

To address the challenge of training our model on a very small dataset, we incorporate the following inductive biases into our transformer encoder layers:

(1.) We replace the fully connected layers in the transformer encoders with sparse autoencoders, an architecture commonly used in neural network interpretability research, because they tend to produce “disentangled” interpretable representations [72]. Intermediate layers in a sparse autoencoder are of much higher dimension than the input layer. An L1 weight penalty, a standard loss penalizing the absolute value of the weights, which encourages sparsity and is also robust to outliers, is applied to ensure that only a small number of neurons remain active. This is thought to create an information bottleneck that promotes efficient encoding and discourages memorization. For simplicity, we apply an L1 loss to all attention weights.

(2.) We add to each attention matrix (a) a positional attention matrix, which does not depend on the patch embeddings but only on their absolute locations in the image to encode the spatial relationships between different patches, regardless of their content, and (b) a localized attention matrix which computes each attention score using an elliptical Gaussian kernel in the two-dimensional space of image patches, with learnable major and minor axes. The positional attention matrix captures global spatial relationships based on the absolute positions of patches, while the localized attention matrix gives more weight to nearby patches with a learnable Gaussian function, with an attention score *L*_*ij*_ between patches *i* and *j* given by:

(1) $$L_{ij\;}∼exp\left(-\frac{{(x}_{i}-x_{j}{)}^{2}}{\sigma_{xi}}-\frac{{(y}_{i}-y_{j}{)}^{2}}{\sigma_{yi}}\right)$$

(where *σ_x_* and *σ_y_* are learnable parameters). Unlike CNNs, which rely on fixed convolutional filters to process local patch interactions, the Gaussian kernel-based attention adapts based on relative positions, allowing for more flexible spatial dependencies. Both positional and localized attention mechanisms require far fewer weights than the full dot-product attention mechanism, at the expense of losing the flexibility and contextual understanding provided by input-dependent attention weights in the full attention mechanism. We apply an L1 penalty on the attention weights to encourage sparse representations, guiding the model to prioritize simple positional attention mechanisms while reducing reliance on more complex global or context-dependent relationships.

The input to the network is a single-channel FAZ OCTA scan of a retinal region that includes the FAZ and the output is a ground truth binary mask of the FAZ boundary, produced by a human annotator. Let *Enc* and *Dec* denote the encoder and decoder models, and let $X\epsilon R^{n\times n}$ be an input image with *n* × *n* pixels, $Y\epsilon {\left\{0,1\right\}}^{n\times n}$ the corresponding ground truth binary mask, of the same dimension, and $\hat{Y}\epsilon {\left[0,1\right]}^{n\times n}$ the output of our model, that is, $\hat{Y}=Dec(Enc(X))$. Each loss term is a weighted sum of binary cross entropy (BCE) loss (a standard loss function for binary classification) and a soft dice loss (another standard loss for segmentation tasks): $L=c_{1}\;L_{BCE}+c_{2}\;L_{Dice}$, where

(2) $$L_{Dice}=1-2\;\frac{\sum_{i}(Y⊙\hat{Y}{)}_{i}+\delta_{1}}{\sum_{i}(Y+\hat{Y}{)}_{i}+\delta_{2}}$$

Here, ⊙ denotes the Hadamard product, and *c*_1_, *c*_2_, *δ*_1_, *δ*_2_ are positive constants (we set *c*_1_ = 1, *c*_2_ = ½, *δ*_1_ = *δ*_2_ = 1). The presence of the dice loss improves model accuracy while the BCE loss improves training stability.

### 2.5. Multi-Condition FAZ Segmentation via Knowledge Distillation for Intra-Modality Data

To enable training on four separate datasets, we employ model distillation. In this technique, separate networks are trained on each dataset, and each specialized “Teacher” network helps train a larger “Base” model. The model distillation literature shows that larger teacher networks can improve the performance of smaller distilled networks and reduce overfitting, especially on small datasets [53]. In our case, the teacher networks are the same size as the distilled network, allowing training over many separate modalities, while each teacher network dedicates all of its parameters to a single condition. Well-trained teacher networks provide richer information to the base model than the target images alone because the former are binary masks while the latter are real-valued probabilities for each pixel.

Our architecture includes the following components: (a) a multi-condition “Base” model, and (b) a series of “Teacher” decoders, each specializing in a single condition (Healthy, AMD, ALZ, or DR). The base model is used across all conditions, while separate teacher decoders are used for each of the four conditions. The encoder is shared across base and teacher models, in order to learn feature embeddings that generalize across conditions. The base and teacher models are trained simultaneously and end-to-end, with gradient information flowing from both base and teacher decodes back through the base encoder. At test time, the teacher decoders are discarded and only the base model is used for inference.

The base decoder receives a one-hot encoding denoting a particular condition, which is projected into the dimension of the image patch feature embeddings using a fully connected network. To account for the possibility that condition information is not available, one can simply add an additional slot to the one-hot encoding and train the base model to make condition-independent predictions as an additional task. We explored several methods for integrating image and condition information, including using attention, but found the most effective approach was to add the condition embedding to each patch embedding and let the attention mechanism learn how best to integrate them. Our architecture is shown in Figure 3.

The loss function for condition *j* includes three terms:

(3) $$L_{j}=\alpha L({\hat{Y}}_{base\;},Y)+\beta L({\hat{Y}}_{teacher\;j\;},Y)+\gamma ({\hat{Y}}_{base\;},Y_{teacher\;j})$$

where the first term compares the output of the base model to the ground truth image, the second term compares the output of the *j*th teacher model to ground truth, and the third term compares the outputs of the base model and *j*th teacher model. Here, *α*, *β*, *γ* are positive constant hyperparameters, with *α* > *β* > *γ* (we set *α* = 1, *β* = ½, *γ* = ¼), so that the model prioritizes the base model over the teacher model, and the fidelity to the ground truth over the comparison between models.

Information flow between the teacher networks and the base network is limited by the fact that gradients must flow through both networks from end to end, which means there is no means of comparing internal representations directly. This limitation can be addressed by comparing the outputs of the networks layer by layer. We add terms to the loss function that mask out residual connections upstream of a given layer, forcing the model to represent as much of the output as possible in the lower layers. Since the size of the layers decreases with depth, the inclusion of these loss terms should force the network to produce compressed representations at each layer, which we expect to generalize better. Hence, our total loss for condition *j* becomes:

(4) $$L_{j,\;tot}=\sum_{i}a_{i}L(Dec_{<i}(Enc(Y)),Y)$$

where $Dec_{<i}$ denotes that every residual layer above layer *i* in our decoder model is masked out. Here, each *a_i_* is a positive constant, with *a_i_* < *a_j_* for *i* > *j* so that the loss prioritizes more complete models. We set $a_{i}=2^{-i}$ so that each term is greater than the sum of all subsequent terms. The single-condition model has 8 attention heads, while the multi-condition model is somewhat larger (16 attention heads) to capture relationships across the four datasets.

### 2.6. Knowledge Distillation for Inter-Modality Data

**Figure 4:**
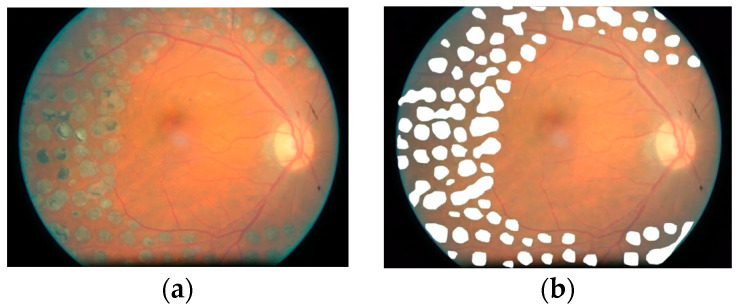
(**a**) Fundus image of the retina in a patient undergoing laser PC therapy. (**b**) Fundus image with masks highlighting the areas treated with laser PC.

Due to the small size of our OCTA FAZ dataset, we explore pretraining our models on a medical segmentation dataset of fundus images from patients undergoing laser photocoagulation (PC) therapy, a specialized ophthalmic procedure involving laser treatment for conditions like diabetic retinopathy. The dataset consists of 298 images, with masks of annotated regions where laser treatment has been applied. An example image from this dataset is pictured in Figure 4.

Pretraining provides the model with a foundation for recognizing structural and tissue distinctions within retinal images, enabling it to handle diverse medical imaging tasks. Color fundus images, despite being from a different domain, capture fundamental retinal features—such as tissue structures, vessels, and abnormalities—shared across ophthalmic conditions. These features help the model learn generalizable representations for segmenting the FAZ in OCTA images, and valuable context for differentiating between normal and pathological structures [73,74,75]. Many large datasets of fundus images are publicly available, making them a good choice for pretraining foundation models [76,77].

Our multi-condition model also supports training using multiple ground truth annotations per image. By interpolating between different annotations, the dataset can be effectively expanded, mitigating systematic biases by some annotators. For instance, when masks by two annotators are available for each image, we can use an expanded one-hot encoding to represent each condition-annotator pair and provide the model with linear combinations of the images and one-hot encodings.

## 3. Results

**Figure 5:**
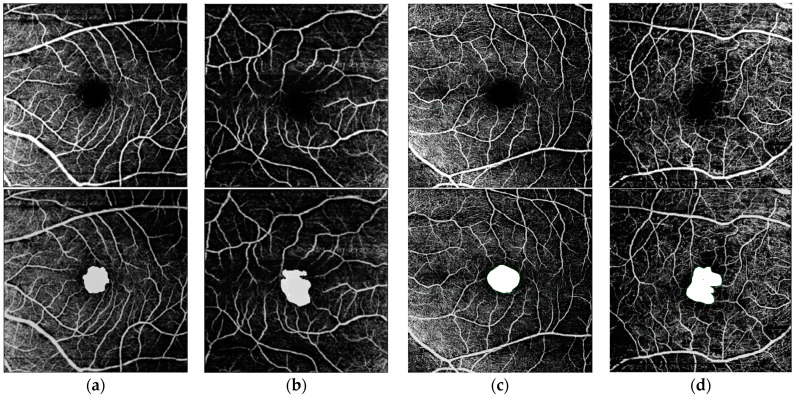
Predicted masks, overlaid on OCTA scans of SVC layer of FAZ, for each of four conditions: (**a**) Healthy (**b**) ALZ (**c**) AMD (**d**) DR. **First Row:** OCTA scan of SVC layer of FAZ. **Second Row:** Predicted binary masks.

The multi-condition model has 8 attention heads and embedding dimensions of 128, 256, and 512 in subsequent layers. We use the Adam optimizer with a learning rate of 1E-4. The input and output data are each tensors of dimension *N_B_* × *N_C_* × *N_H_* × *N_W_*, together with a one-hot-encoding vector of size *N_B_* × *N_D_*, where *N_B_* is the batch size, *N_C_* is the channel dimension, *N_H_* and *N_W_* are the image height and width, and *N_D_* is the number of conditions. (We set: *N_B_* = 10, *N_C_* = 1, *N_H_* = *N_W_* = 256, *N_D_* = 4.) We cycle through conditions during training, training each for one epoch at a time. Data augmentation included random shifts, rotations, flips, color jitter, and additive zero-mean Gaussian white noise. The model was trained for 500 epochs, approximately 24 hours on one NVIDIA GeForce RTX 3090 GPU. The model is evaluated using two-fold cross-validation (training on one set and testing on the other, then swapping the sets), with the same random partition applied to each condition. This process is repeated for each condition to ensure a robust evaluation across all datasets. Example model inputs/outputs for each condition are shown in Figure 5.

The following tables show two-fold cross validation dice indices on the test set for single-condition and multi-condition models across different retinal conditions. Table 1 shows the effect of PC pretraining on single-condition and multi-condition model performance for the Healthy, ALZ, AMD, and DR datasets. We pretrain both our base and teacher models on PC color fundus images and then finetune on our FAZ dataset. Table 2 compares the effect of using two annotations during training under the same pretraining conditions. As the small size of the test set results in somewhat variable performance, we compute an average of the test accuracy over the final 100 training epochs, providing a more stable measure of model performance. The average test dice score on the single-condition PC pretraining data is 52.5%.

The results in Table 1 show that multi-condition models typically outperform single-condition models. Notably, multi-condition models benefit the most from PC pretraining, achieving the highest overall mean dice index, with consistent improvements across all conditions but AMD. Although single-condition models also show slight improvements with pretraining, the gains are smaller, suggesting that multi-condition models more effectively leverage pretraining to enhance generalization.

While PC pretraining enhances performance for healthy and DR cases in both single-condition and multi-condition models, it appears to have no significant effect on ALZ segmentation and a negative effect on AMD segmentation, particularly in the multi-condition model. This decline suggests that the pretraining may introduce domain-specific biases that are less effective for certain pathological features, which suggests the need for targeted adjustments to pretraining or changes to model architecture to mitigate negative knowledge transfer. Additionally, the smaller gains from pretraining in single-condition models imply that multi-condition configurations are better suited for transferring learned features across diverse conditions. The largest improvement in PC pretraining is observed in healthy eyes (the smallest dataset).

To further analyze model performance, a paired *t*-test was conducted to assess the significance of the observed improvements on the test data over the final 100 epochs of training (Table 3). A paired *t*-test is appropriate in this case because it compares the mean differences in performance between two conditions (e.g. pretraining vs. no pretraining) for the same set of models. This test controls for individual model variability by focusing on the differences within paired observations, allowing us to assess whether the observed improvements are statistically significant. We also calculated Cohen’s *d*-values, which measure the effect size (Table 4).

PC pretraining and the inclusion of two annotations showed large effect sizes for Healthy eyes in both single-condition and multi-condition models but exhibited variable effects across conditions, with notable improvements in DR and moderate gains in ALZ. In contrast, the AMD dataset experienced mixed results, including, in one case, a small decline in performance, likely due to negative knowledge transfer or the presence of domain-specific biases that the model failed to adequately address. This indicates that AMD’s unique pathological features may conflict with the shared features learned across conditions in the multi-condition approach, which suggests the need for further refinement in the way the model represents shared features, a greater ability to specialize to conditions, and targeted pretraining strategies.

The relatively small improvement from multi-condition training suggests that the differences in the FAZ between these conditions is relatively small. The small improvement from pretraining on PC fundus images, and slightly worse performance for AMD, suggests that the general features of retinal structure learned from this data are of limited value, and more specialized FAZ data is necessary to improve performance. Inconsistency or ambiguity in FAZ annotations also limits performance.

## 4. Discussion and Conclusions

In this study, we presented a multi-condition transformer-based architecture designed to improve automated segmentation of the FAZ in a variety of eye diseases, such as AMD, ALZ, and DR. Our approach addresses the challenges posed by limited and imbalanced datasets by leveraging knowledge distillation from multiple retinal diseases. The results demonstrate substantial improvements in segmentation performance in scenarios with very small training sets, and the model’s ability to generalize across different diseases. The multi-condition model generally outperformed single-condition models, demonstrating its ability to leverage shared features across conditions, though certain pathological features in AMD require further attention.

Adaptive pretraining strategies, additional methods of selective feature sharing, or condition-specific regularization could mitigate domain-specific biases seen in AMD, while tailored data augmentation strategies and synthetic data generation techniques that simulate condition-relevant pathological variations could provide more representative training data.

The proposed FAZ segmentation framework may be of use in the study of systemic diseases that manifest through retinal vascular abnormalities, such as diabetes, hypertension, and neurodegenerative disorders like ALZ, including for identifying biomarkers for early detection, disease progression monitoring, and treatment efficacy assessment. Large-scale implementation of such models could facilitate population-level screening and early intervention for high-risk groups, supporting public health initiatives and epidemiological research. Integrating this framework with other diagnostic tools, such as AI-based fundus analysis or blood biomarker studies, could enhance clinical workflows.

Extensions of this work could focus on cross-modal integration with other imaging techniques, combining OCTA with techniques such as fundus photography or fluorescein angiography. Expanding the applications of the model to include a wider range of systemic conditions would increase its utility in medical practice and research. Multi-condition segmentation methods could also be combined with next-frame prediction techniques for longitudinal data to track disease progression over time.

## Figures and Tables

**Table 1:** Two-fold cross-validation dice indices of trained models.

	Model	Healthy	ALZ	AMD	DR	Mean
Single-condition Model	Without pretraining	86.4	80.6	82.8	79.6	82.0
	With pretraining	86.6	83.1	82.8	80.8	83.1
Multi-condition Model	Without pretraining	85.4	83.7	82.9	83.3	83.7
	With pretraining	88.0	83.9	81.3	84.4	83.8

**Table 2:** Two-fold cross-validation dice indices of model trained using two annotations.

	Model	Healthy	ALZ	AMD	DR	Mean
Single-condition Model	Without pretraining	85.3	81.3	83.9	81.2	81.1
	With pretraining	86.4	84.1	85.4	81.0	84.2
Multi-condition Model	Without pretraining	86.2	84.0	82.3	82.5	83.5
	With pretraining	87.1	84.1	82.3	83.3	83.9

**Table 3:** Paired *t*-test *p*-values comparing model performance. (<0.05: reject null hypothesis).

	Model	Healthy	ALZ	AMD	DR
Single-condition Model	With/without pretraining	0.005	<0.001	0.94	<0.001
	With/without 2 annotations	<0.001	0.006	<0.001	<0.001
Multi-condition Model	With/without pretraining	<0.001	0.21	<0.001	<0.001
	With/without 2 annotations	<0.001	0.09	0.03	0.001
Single-condition/Multi-condition Model		<0.001	<0.001	<0.001	<0.001

**Table 4:** Cohen’s *d*-threshold. (|*d*|: small: ≤ 0.2, medium: 0.5, large: ≥ 0.8).

	Model	Healthy	ALZ	AMD	DR
Single-condition Model	With/without pretraining	0.3	1.8	0.0	0.86
	With/without 2 annotations	−1.1	0.3	0.8	1.2
Multi-condition Model	With/without pretraining	3.2	0.1	−0.7	0.6
	With/without 2 annotations	0.6	0.2	−0.2	−0.3
Single-condition/Multi-condition Model		1.7	2.6	1.3	0.5

## Data Availability

The datasets are available from the corresponding author on reasonable request. The code generated during the study is accessible from the corresponding author based on reasonable request and subject to the regulations of the institute.
